# Preclinical Evidence for the Role of Botulinum Neurotoxin A (BoNT/A) in the Treatment of Peripheral Nerve Injury

**DOI:** 10.3390/microorganisms10050886

**Published:** 2022-04-24

**Authors:** Michael Adler, Sabine Pellett, Shashi K. Sharma, Frank J. Lebeda, Zygmunt F. Dembek, Mark A. Mahan

**Affiliations:** 1 Neuroscience Department, Medical Toxicology Division, U.S. Army Medical Research Institute of Chemical Defense, 8350 Ricketts Point Rd., Aberdeen Proving Ground, MD 21010, USA; 2 Department of Bacteriology, University of Wisconsin-Madison, 1550 Linden Drive, Madison, WI 53706, USA; sabine.pellett@wisc.edu; 3 Division of Microbiology, Center for Food Safety and Applied Nutrition, Food and Drug Administration, College Park, MD 20740, USA; shashi.sharma@fda.hhs.gov; 4 Biotechnology, Protein Bioinformatics, Zanvyl Krieger School of Arts & Sciences, Johns Hopkins University, Advanced Academic Programs, 9601 Medical Center Drive, Rockville, MD 20850, USA; fjl525@aol.com; 5 Department of Military and Emergency Medicine, Uniformed Services University of Health Sciences, 3154 Jones Bridge Rd., Bethesda, MD 20814, USA; zfdembek@gmail.com; 6 Department of Neurosurgery, Clinical Neurosciences, University of Utah, 175 N Medical Drive East, Salt Lake City, UT 84132, USA; mark.mahan@hsc.utah.edu

**Keywords:** botulinum neurotoxin A, BoNT/A, conditioning lesion, chronic constriction injury, crush injury, peripheral nerve injury, PNI, nerve regeneration, reinnervation, Schwann cells, angiogenesis

## Abstract

Traumatic peripheral nerve injuries tend to be more common in younger, working age populations and can lead to long-lasting disability. Peripheral nerves have an impressive capacity to regenerate; however, successful recovery after injury depends on a number of factors including the mechanism and severity of the trauma, the distance from injury to the reinnervation target, connective tissue sheath integrity, and delay between injury and treatment. Even though modern surgical procedures have greatly improved the success rate, many peripheral nerve injuries still culminate in persistent neuropathic pain and incomplete functional recovery. Recent studies in animals suggest that botulinum neurotoxin A (BoNT/A) can accelerate nerve regeneration and improve functional recovery after injury to peripheral nerves. Possible mechanisms of BoNT/A action include activation or proliferation of support cells (Schwann cells, mast cells, and macrophages), increased angiogenesis, and improvement of blood flow to regenerating nerves.

## 1. Introduction

The peripheral nervous system (PNS) consists of sensory, motor, and autonomic nerve fibers, support cells, and ganglia [1]. Peripheral nerves serve as essential input and output communication links between the CNS and target tissues. Their vulnerable location on the superficial surfaces of limbs and flexible joints renders peripheral nerves highly susceptible to traumatic injuries [1,2,3]. In addition to trauma, peripheral nerve injury (PNI) may result from compression, ischemia, tumors, electric shock, temperature extremes, surgery, entrapment syndromes, and inherited or acquired neuropathies [3,4,5]. Serious injuries to peripheral nerves can lead to impaired sensory, motor, and autonomic function, and are often accompanied by pain, such as tactile allodynia and sensations of burning, tingling, or numbness in the injured tissue [2,6,7].

In non-combat situations, the most severe forms of PNI are generally associated with vehicular collisions, especially those involving motorcycles, as well as from penetrating trauma, such as falls and industrial accidents. PNI affects young people of working age disproportionately, limiting their quality of life and reducing their ability to fully engage in the workforce [4,8,9]. In the U.S., the number of patients admitted yearly to a hospital for treatment of a PNI is approximately 57 per million (0.0057%); however, these injuries account for approximately 3% of patients admitted to Level I trauma centers [10,11]. PNIs exert a disproportionate socioeconomic burden to society because of the extensive critical care and rehabilitation resources needed during the acute phase of injury and for long-term disability care that may be required, in some cases for a lifetime [9,12].

Under wartime conditions, wounds to the periphery from shrapnel, bullets, bombs, and other explosive devices are the most common sources of nerve trauma [2,13]. The frequency of PNIs among warfighters engaged in combat is considerably higher than the incidence of PNI in the general population (8% versus 3%), and the injuries tend to be more severe [13]. Wounds to the extremities (which are not protected by modern armor) are the most frequent cause of permanent disability in warfighters [14,15], and these often involve PNI in the affected limbs [16]. In a study of British combatants injured in Iraq and Afghanistan between 2003 and 2014, 77% sustained injury to an extremity, and 11% required at least one amputation at the level of or proximal to the wrist or ankle [17].

Peripheral nerve injuries were first studied systematically during the U.S. Civil War (1860–1864). War-related injuries and diseases over the next century and a half served as the catalysts for improvements in the treatment of nerve injuries, infectious diseases, wound care, orthopedic and plastic surgery, and rehabilitation medicine [2,14,17,18]. During World War I, military and civilian surgeons established the fundamental techniques for nerve repair consisting of “mobilization of the nerve stump, resection back to healthy bundles centrally and peripherally, and end-to-end suture” (neurorrhaphy) [19]. This approach is still considered the standard for nerve repair when the proximal and distal ends can be coapted without tension [4,5,12].

The severity, location, and other characteristics of the PNI determine the potential of nerves to recover through genetically programmed physiological processes. In his classic 1943 publication entitled “Three types of nerve injury,” the famed neurosurgeon Herbert Seddon classified nerve injuries into three categories based on their natural history [20]. The least severe was termed neurapraxia, which is typically caused by compression and leads to demyelination at the site of injury without loss of axonal continuity or disruption of the connective tissue sheaths. Neurapraxia is characterized by transient muscle weakness and loss of sensation, but usually resolves spontaneously. Next in severity is axonotmesis, which results from more severe trauma (such as nerve crush) that disrupts the axon and myelin sheath, but largely spares neuronal connective tissues, thus still allowing for spontaneous recovery. The most severe injury is neurotmesis, defined by Seddon as a complete severance of the nerve plus disruption of neural connective tissues; recovery from neurotmesis is not possible unless the nerve is surgically repaired. In 1951, Sidney Sunderland [21] expanded this classification scheme to five grades of severity within Seddon’s overall conceptual framework. Sunderland retained the categories of neurapraxia and neurotmesis, but subdivided axonotmesis into three degrees of severity based on the extent of connective tissue injury. The Seddon and Sunderland classifications are still in current use, and provide guidance for surgical intervention and optimal care.

The innate capacity of peripheral nerves to regenerate diminishes if treatment is not promptly initiated [22]; this was a key lesson learned in dealing with combat-related PNIs during World War II [13]. The reduction in successful reinnervation stems from the loss of the growth-supporting environment of the severed distal nerve stump and to the development of atrophy and fibrosis in denervated muscles [22,23]. Reinnervation is also less successful with longer nerves, because the slow rate of regeneration (1–3 mm/day in humans) makes it difficult for these nerves to reach their distant targets before the environment necessary to support and guide the regenerating axon is lost [4,23]. In addition, misdirection of growing neurites frequently leads to failed reinnervation in proximal injuries or in nerves that coordinate complex movements. Consequently, recovery from severe PNI is often incomplete, leading to substantial and persistent functional disability [22,23,24]. To exploit the critical time window for successful recovery, development of therapeutic agents to accelerate the rate and accuracy of reinnervation would be desirable [3]. During the last decade, numerous candidates have been examined, almost all in animal studies, including corticosteroids [25], the immunosuppressant drug FK-506 [26], insulin-like growth factor-1 (IGF-1) [27], vascular endothelial growth factor (VEGF) [28], transforming growth factor-β1 (TGF-β1) [29], brain-derived neurotrophic factor (BDNF) [30], 4-aminopyridine (4-AP) [31], and botulinum neurotoxin A (BoNT/A) [30,32]. A comprehensive discussion of drug candidates can be found in a recent review by Bota and Fodor [33]. The current review will focus on the ability of BoNT/A to accelerate nerve regeneration and on its potential to aid in the treatment of PNI.

The mechanism of BoNT/A in enhancing recovery from PNI remains to be established; however, it appears to be largely distinct from its well-known paralytic action on smooth and skeletal muscle. Possibilities include increased activation/proliferation of support cells, especially Schwann cells, inhibition of vasoconstrictor neurons leading to improved blood flow and increased angiogenesis [1,34]. The latter suggestion is consistent with the prominent role of angiogenesis in wound healing [35,36,37] and with the findings that BoNT/A can relieve vasospasm due to frostbite [38], Raynaud’s phenomenon [39] or ischemic injury to the digits [40].

Since BoNT/A has been used with considerable success for treatment of neurological and autonomic dysfunction [41,42], migraines [43], a variety of pain conditions [44,45], and for aesthetic improvements [46], it would be of considerable interest to determine whether BoNT/A could also be of benefit in the treatment of PNI. Accordingly, our aim was to review the relevant and especially recent literature on the ability of BoNT/A to accelerate recovery from PNI in animal models and to assess the potential role of this toxin for improving the outcome of PNI in humans. Related topics such as the role of BoNT in the treatment of neuropathic pain will only be dealt with briefly, since they have been covered thoroughly elsewhere [44,47,48]. For a recent review on the effects of BoNT on injuries to the nervous system that includes the CNS, the reader is referred to Luvisetto [49].

We searched PubMed and Web of Science to identify studies published between January 2000 and February 2022 involving BoNT and PNI. The following search statements were used: ((botulinum neurotoxins AND (peripheral nerves OR peripheral nervous system)) AND (peripheral nerve injuries OR crush injuries OR vasospastic disorder OR frostbite OR recovery of function). In addition, we examined articles cross-referenced from research papers and reviews that were identified from our search strategy. The search returned 184 articles, whose abstracts were screened by two authors for duplication and relevance. Studies dealing with Clostridium botulinum C2 or C3 toxins were excluded since their mechanisms of action differ from those of the botulinum neurotoxins [50]. Nineteen articles were deemed to be relevant to this review: 18 of these focused on BoNT/A, and 1 dealt primarily with BoNT/B. The studies indicate that BoNT/A can increase nerve regeneration rates and improve sensory and motor function after PNI in mice and rats. Since the number of publications on the primary topic was limited, this review will be largely descriptive.

## 2. Organization of the PNS

The PNS has two major subdivisions, autonomic and somatic, which together encompass the entire nervous system outside the brain and spinal cord. The somatic subdivision consists of sensory (afferent) and motor (efferent) axons. Afferent fibers have their cell bodies in the dorsal root ganglion and convey sensory information from the periphery to the central nervous system (CNS). Efferent fibers have their cell bodies in the ventral horn of the spinal cord and innervate skeletal muscle to control muscle tone and voluntary movement [51]. Peripheral nerves consist of axons, support cells, blood vessels, and connective tissues; the latter protects delicate axons from trauma and provides the scaffold required for successful regeneration after injury [52]. The importance of the intact nature of this scaffold cannot be underestimated since most of the success of repair after PNI in humans depends on the extent of damage to the nerve sheath (to be discussed later). The connective tissues are arranged in three concentric compartments consisting of (innermost to outermost) the endoneurium, the perineurium, and the epineurium. The endoneurium is the honeycomb-like core of the nerve containing the endoneurial tubes enclosing myelinated axons, Remak bundles (nonmyelinated axons), and resident macrophages and fibroblasts [53]. Nerve fibers within the endoneurium are bathed in endoneurial fluid, which is similar in function to cerebrospinal fluid [54]. The endoneurial capillaries have properties analogous to those that contribute to the blood–brain barrier in the CNS, termed the blood-nerve barrier (BNB), which restrict certain substances in blood (e.g., macromolecules, hydrophilic molecules) from entering the endoneurial fluid [55]. Pathological alterations in endoneurial capillary structure (e.g., loss of pericytes, basement membrane thickening, or endothelial hyperplasia) may be a key trigger in peripheral neuropathies such as diabetic neuropathy [56]. The perineurium also serves as part of the BNB and divides bundles of axons into fascicles in large mammalian polyfascicular nerves [57,58]. In addition, the perineurium is intimately involved in peripheral nerve regeneration [59,60,61,62]. The epineurium binds individual fascicles (interfascicular epineurium) and forms the outermost connective tissue sheath (external epineurium) [63].

### 2.1. The Autonomic Nervous System (ANS)

The ANS consists of three branches, sympathetic, parasympathetic, and enteric, as originally described by the renowned Cambridge University physiologist John Langley in 1898 [64]; these branches work in concert to maintain physiological homeostasis in the face of internal and external challenges [65,66]. The enteric branch contains sensory neurons and interneurons in addition to motor neurons and regulates the function of the digestive tract [67]. The sympathetic and parasympathetic branches consist of efferent (motor) axons that innervate the heart, smooth muscles, and secretory glands and afferent (sensory) neurons that project either directly to autonomic ganglia or to the CNS. The former allows for local reflex control, while the latter enables integration and modulation of postganglionic activity by the CNS.

The major center of integration for the ANS is the hypothalamus, which receives input from higher cortical centers via the limbic system and from peripheral organs via sensory autonomic fibers. The most important hypothalamic nucleus in this regard is the paraventricular (PVN) [65,66]. The PVN receives direct sympathetic and parasympathetic afferent inputs from the trigeminal pars caudalis and the nucleus tractus solitarius, respectively.

As with somatic nerves, damage to autonomic nerves can result in loss of control of vital functions. Depending on the location and nature of the injury, these may involve impaired cardiovascular control, excessive or insufficient sweating (leading to poor temperature control), difficulty swallowing, nausea, vomiting, diarrhea or constipation, problems with urination, abnormal pupil size, and sexual dysfunction. Of particular interest for the current review is the control of blood flow in peripheral nerves by sympathetic vasomotor fibers that innervate vascular smooth muscle [68]. Release of norepinephrine (NE) and co-transmitters from these nerve endings leads to vasoconstriction and reduction in blood flow; thus, inhibition of neurotransmitter release by BoNT/A may account, in part, for the beneficial effects of BoNT/A in accelerating the regeneration rate of axons following PNI [1,35,36,37,38,39,40].

### 2.2. Role of Myelin in Nerve Conduction

Neurons in both the PNS and CNS possess voltage gated ion channels, and their axons have the unique ability to propagate action potentials over considerable distances at constant amplitude and velocity [69]. Without the precise arrangement and distribution of these ion channels, action potentials would decrement with distance and fail to reach their targets. The conduction velocity of axons is determined by their fiber diameter but can be greatly enhanced by the presence of myelin, an insulating sheath produced by Schwann cells [69]. Myelin is able to perform this function by shielding axons from the interstitial fluid and forcing the ionic currents responsible for the action potential to flow predominantly through the low resistance pathway of the axoplasm. These currents cross the high resistance axonal membrane only at the nodes of Ranvier, which are periodic gaps in the myelin sheath where ionic currents are regenerated [70,71].

In myelinated nerve fibers, the Na^+^ channels responsible for propagation of the action potential are concentrated at these nodes, but in nonmyelinated fibers, they are distributed throughout the axon [69]. The presence of myelin enables axon conduction velocities to reach up to 120 m/s, whereas nonmyelinated axons are limited to conduction velocities of 0.5 to 10 m/s. Rapid impulse transmission is important since it determines the rate of information flow and processing in the nervous system [71]. Another essential role of Schwann cells is to support axonal regeneration following injury by undergoing conversion to a repair-promoting phenotype as described in the next section [72].

### 2.3. Sequelae Following Traumatic Injury to Peripheral Nerves

Injury to peripheral nerves can result in impaired sensation, muscle weakness, and, in severe cases, in extensive muscle atrophy. Unlike axons in the CNS, those in the PNS have a high intrinsic ability to regenerate, due in part to the regenerative environment created by Schwann cells [72,73,74]. Trauma to peripheral nerves such as crush or axotomy leads to marked alterations in the axon proximal and distal to the site of injury, and to chromatolysis and other adaptive changes in the soma [51]. Chromatolysis reflects an underlying disaggregation and redistribution of rough endoplasmic reticulum and polyribosomes (Nissl substance) to enable regeneration of the injured axon, which requires a shift from synthesis of neurotransmitters, receptors, and ion channels to the production of molecules needed for nerve regeneration such as neurofilaments and other cytoskeletal components [75,76]. Additional changes in the soma in response to axonal injury include enlargement and peripheral displacement of the nucleus, increase in the volume of the soma and retraction of dendrites [76]. Nerve injuries that occur close to the soma have a low probability of successful resolution, often culminating in death of the neuron [51]. Degeneration of the axon distal to the injury is believed to be an intrinsic response of the injured axon [77].

Successful reinnervation following traumatic nerve injury requires coordination between neurons and support cells, especially Schwann cells and macrophages [78]. In response to the injury, Schwann cells undergo conversion from a myelin-producing phenotype to a repair-promoting phenotype (repair Schwann cells) under the primary control of the transcription factor c-Jun [72]. These modified Schwann cells play a key role in promoting the survival of peripheral nerves after injury and for enabling peripheral nerves to reinnervate their targets [34].

Complete recovery of sensory and motor function is more likely for injuries caused by crush than for those involving transection, because the former preserves the connective tissue scaffold that the regenerating axons need to reach peripheral targets [1,20,21,78]. With either mode of injury, however, the axon becomes separated from the cell body and no longer transmits impulses between the CNS and periphery [22]. This is also the case with milder crush, where there is extensive loss of myelinated large diameter Aα and Aβ axons [79]. To restore function, the axon segments distal to the injury must undergo degradation and removal. This is carried out by a process termed Wallerian degeneration (WD), named for the eminent British neuroanatomist Augustus Waller who first described the phenomenon in the frog glossopharyngeal and hypoglossal nerves as “alterations which take place in the elementary fibers of the nerve after they have been removed from their connection with the brain and spinal marrow” [80]. WD was initially considered to be a passive phenomenon, but with increasing understanding of its molecular controls, WD has come to be recognized as an ordered process involving a complex interplay of cells and mediators to create a microenvironment that allows successful regrowth of axons from the proximal nerve segment [81,82].

Degeneration of the distal segment is an essential first step in this process, since the injured distal stump would impede reinnervation if it maintained its connection with its peripheral target. Degeneration of axons involves calpain-mediated proteolysis of neurofilaments and other structural proteins, causing damaged axons along with their myelin sheaths to break up into ellipsoids [83,84]. Removal of the ellipsoids is accomplished by recruitment of activated macrophages and Schwann cells into endoneurial tubes to phagocytose these and other cellular debris [73,85,86,87,88]. Thereafter, Schwann cells align and form columns called bands of Büngner, which create a scaffold for guiding axonal regeneration [74]. The region of nerve proximal to the trauma undergoes dieback until encountering the first node of Ranvier outside the zone of injury. Degeneration of the proximal stump then terminates and regeneration begins by formation and elongation of growth cones at the proximal nerve stump [1,63]. Neuron regeneration is most efficient proximal to the injury site; Schwann cells distal to the injury site lose their ability to induce nerve regeneration with distance and time, leading to incomplete recovery [22,88,89,90,91]. Thus, treatments that can bring about rapid induction of neuronal repair mechanisms hold considerable promise for improving the outcome of PNI.

## 3. Attributes of BoNT/A That Make a Promising Therapeutic Agent for Treatment of PNI

Although substantial advances in surgical procedures have and continue to be made [4], recovery of normal function after nerve injury is often elusive, especially when the injury is close to the spinal cord, and regeneration of the injured nerve over long distances is required to achieve reinnervation of the target [30,74]. Physical therapy is integral to optimal recovery, yet the pain associated with injury or surgery may limit the ability of patients to perform the required exercises. In the search for a pharmacological treatment, BoNT/A is a reasonable candidate to improve the outcome of peripheral nerve injuries based on its selectivity [92,93], persistence [94,95], safety [96], and success in the treatment of conditions such as muscle hyperactivity [42], migraine [43], and neuropathic pain [44,45,47,48]. BoNTs comprise a large family of protein toxins secreted primarily by Clostridium botulinum, a gram positive, rod-shaped, spore-forming anaerobic bacterium. Seven serotypes, designated A-G, are currently known, and recent progress in microbial genome sequencing has identified numerous subtypes, chimeric toxins and BoNT-like putative toxins, each with unique properties which may find future therapeutic use [97,98,99,100].

These neurotoxins have historically been associated with food poisoning and are designated as Tier 1 Select Agents by the U.S. Department of Health and Human Services [101,102] for their potential to be used as bioterrorism agents. However, the overwhelming interest in the BoNTs, since the initial approval of serotype A by the U.S. Food and Drug Administration (FDA) in 1989, stems from their benefit for treatment of an increasing number of medical conditions; since 2002, use of BoNT has also extended to facial aesthetics [103]. Currently, only BoNT/A1 (onabotulinumtoxinA, Botox^®^; abobotulinumtoxinA, Dysport^®^; incobotulinumtoxinA, Xeomin^®^), and BoNT/B1 (rimabotulinumtoxinB, MyoBloc^®^/NeuroBloc^®^) are approved by the FDA for human use [104,105]. BoNT/A1 and BoNT/B1 share a broadly similar mode of action, and exposure to either serotype leads to cleavage of a SNARE (soluble-N-ethylmaleimide sensitive factor attachment protein receptor) protein: SNAP-25 in the case of BoNT/A1 and vesicle-associated membrane protein 1/2 (VAMP 1/2) in the case of BoNT/B1. SNARE protein cleavage leads to inhibition of transmitter release at the neuromuscular and neuroeffector junctions, culminating in inhibition of the target muscle, secretory gland, or nociceptive pathway. However, the serotypes differ considerably in potency, nerve terminal binding sites, persistence, and therapeutic index. BoNT/A1 is more potent, has a longer duration of action and exhibits a greater margin of safety [106]. In the U.S., BoNT/A1 is approved for the treatment of strabismus, blepharospasm, hemifacial spasm, cervical dystonia, moderate to severe glabellar lines, axillary hyperhidrosis, chronic migraine, headache, urinary incontinence, crow’s feet, and upper limb spasticity. BoNT/B1 is approved for two indications: cervical dystonia and chronic sialorrhea [105,107]. In addition to the approved indications, BoNT/A1 has an even greater number of off-label uses including treatment of chronic and neuropathic pain [44,48,108]. A complete list of indications for Botox^®^ (onabotulinumtoxinA) can be found on the Allergan website [109].

The role of BoNT in pain management was originally attributed to its ability to block ACh release with subsequent relaxation of abnormally contracting muscles, based on the widely held belief that BoNTs mainly target peripheral motor nerve endings while largely sparing sensory and CNS neurons. However, it has been demonstrated that BoNTs can block transmitter release in essentially all neurons (as well as in some non-neuronal cells), although generally at higher concentrations [110,111,112]. The current view is that the mechanism of BoNT in alleviating pain is complex and involves inhibition of pain mediators such as substance P, calcitonin gene-related peptide (CGRP), and glutamate [113,114,115]. Moreover, the antinociceptive action of BoNT/A may involve retrograde transport to the CNS and transcytosis to second order neurons [116]. Although evidence continues to accumulate for retrograde transport of BoNT/A [114,115], evidence for transcytosis following peripheral administration of BoNT/A is less compelling [117].

Muscle relaxation also contributes to relief of pain by increasing blood flow and releasing nerve fibers from compression of abnormally contracting muscle; this, however, does not appear to be the primary mechanism for the analgesic effect of BoNT/A [118]. Interestingly, authors studying the antinociceptive properties of BoNT/A in rodent models have generally not reported muscle weakness at doses and routes of administration that produce analgesia, which appears to be at odds with motor nerve terminals being the dominant site of BoNT/A action [119]. This may indicate the importance of targeting the toxin to a specific injection site to limit uptake by motor nerve endings. In humans treated with BoNT/A for neuropathic or non-neuropathic pain, the incidence of muscle weakness as an adverse event, is exceedingly rare [47,48].

### 3.1. BoNT/A Enhances Recovery from PNI in Chronic Constriction Injury and Nerve Crush Models in Mice and Rats

In contrast to the large body of evidence on the analgesic effects of BoNT/A [118], the ability of botulinum neurotoxins to accelerate regeneration rates and to enhance recovery of function after traumatic injury of peripheral nerves is less well known. The regenerative potential of BoNT/A was not appreciated until relatively recently, perhaps because initial clinical indications for BoNT/A focused largely on its ability to inhibit abnormal muscle tone [42,105].

The ability of BoNT/A to improve functional recovery from peripheral nerve injury was first reported by Marinelli et al. [120]. The authors used the chronic constriction injury (CCI) model of Bennett and Xie [121] to cause partial injury of the sciatic nerve in mice and rats proximal to its trifurcation into the sural, tibial, and common peroneal nerves. CCI was elicited by tying several ligatures loosely around a segment of the sciatic nerve, resulting in impaired epineural blood flow, inflammation, and intraneural edema. Injury to axons was evident within 24 h after CCI and resulted from the tension exerted on swollen nerves by the rigid ligatures. CCI generated damage similar to that observed in human entrapment neuropathies [21].

CCI impaired both sensory and motor function. Sensory abnormalities included cutaneous allodynia and thermal hyperalgesia (neuropathic pain); motor impairment was evidenced by atrophy of muscles innervated by the sciatic nerve and an inability of the hindlimb on the injured side to bear weight or to exhibit normal walking track characteristics. CCI led to a disproportionate loss of large myelinated nerve fibers, while many unmyelinated C-fibers (important in pain mechanisms) remained intact. Injured C-fibers were noted to exhibit enhanced long-term potentiation (LTP), which may be responsible, at least in part, for the neuropathic pain resulting from this procedure [121].

Marinelli et al. [120] found that a single intraplantar (or intrathecal) injection of BoNT/A (pure 150 kDa neurotoxin) was able to counteract CCI-induced mechanical allodynia and thermal hyperalgesia. The actions of BoNT/A were rapid, dose-dependent, and persistent. These findings confirmed and extended results from earlier studies on the ability of BoNT/A to alleviate neuropathic pain [122,123]. Marinelli et al. [120] also demonstrated that BoNT/A could accelerate functional recovery from CCI in mice. Restoration of function was assessed by (1) the percent of body weight supported by the injured (ipsilateral) and contralateral hindlimbs (measured by the incapacitance test) and (2) the walking track pattern displayed by the two hindlimbs (expressed as the sciatic static index). The latter is similar to the sciatic functional index, but has the advantage of being independent of gait velocity or changes in direction of locomotion [124]. In control mice, each hindlimb bore 50% of the body weight, and each foot showed a characteristic toe spread during locomotion with the rear portion of the paw making minimal contact with the substrate. CCI led to a marked shift of body weight to the contralateral hindlimb and to an abnormal walking pattern characterized by an absence of toe spread, ventroflexion of the toes, and extensive contact with the rear section of the paw. In mice receiving an intraplantar injection of saline, these measures of functional impairment were slow to recover: recovery from abnormal weight bearing required >30 days, and abnormalities of walking pattern were still incomplete at end of the study (81 days after onset of CCI).

In contrast, a single intraplantar injection of a non-paralytic dose of BoNT/A (15 pg) in the injured hindlimb led to a rapid and pronounced improvement in weight bearing in the injected hindlimb; recovery was largely complete within 1 day of BoNT/A administration and was maintained for the duration of the study at values close to those of the contralateral hindlimb. The abnormalities in the walking pattern showed an accelerated but more gradual recovery after intraplantar injection of BoNT/A, with the sciatic static index reaching approximately 70% of control at day 81. BoNT/A also increased the expression of the nerve regeneration marker cyclin-dependent kinase-2 (Cdc2) and of the repair Schwann cell phenotype markers glial fibrillary acidic protein (GFAP) and S100 calcium binding protein β (S100β). The actions of BoNT/A on weight bearing and locomotion, in conjunction with the enhanced expression of the above markers, indicate an accelerated reinnervation of hindlimb muscles. However, the effects of BoNT/A on pain relief may also have contributed to the functional improvements; thus, more direct evidence would be needed to confirm that an increased reinnervation of motor axons was the salient factor responsible for this finding. In addition, since CCI generates incomplete nerve injury, it would be of interest to determine whether the regenerative actions of BoNT/A can also occur under conditions of more severe injury such as nerve crush or transection.

These issues were addressed by Cobianchi et al. [32], who examined the effect of a sub-paralytic dose of BoNT/A (pure 150 kDa neurotoxin) in both nerve crush and CCI models. In the former, the mouse sciatic nerve was crushed by means of fine forceps, and immediately thereafter, either 2 µL BoNT/A (15 pg) or saline was injected into the nerve at the site of injury. The effects of intraneural administration of BoNT/A were examined as a function of time after the injury on several indicators of recovery including axonal growth rates, the number and density of regenerated nerve fibers and the amplitude and latency of compound muscle action potentials (CMAPs).

The major findings were that BoNT/A injection led to a significant increase in the rate of reinnervation of myelinated neurofilament 200+ (NF200+) axons in motor and sensory nerves but had no effect on presumptive non-myelinated peptidergic sensory CGRP+ nerve fibers [125]. The reinnervation rate for NF200+ fibers was 3.2 mm/day in the BoNT/A-injected mice vs. 2.8 mm/day in saline-injected mice, which led to a difference in length of 16.3 mm vs. 14 mm, respectively, at day 7 after nerve crush [32]. In addition to a more rapid rate of axonal growth, the regenerative effect of BoNT/A was also demonstrated in histological studies that revealed a significant increase in the number (28%) and density (44%) of myelinated fibers at day 14 after crush. A functional effect of BoNT/A on electrophysiological parameters was inferred by findings that the amplitude of CMAPs in gastrocnemius and plantar muscles, both innervated by the tibial division of sciatic nerve, was elevated at day 14 after crush in BoNT/A-injected mice but not in mice injected with saline. However, since the values did not reach significance, this finding must be interpreted with caution.

In the CCI model, intraplantar injections of 20 µL BoNT/A (15 pg) or 20 µL saline were made in the ipsilateral paw 5 days after sciatic nerve ligation. The effect of BoNT/A was evaluated by eliciting CMAPs in the gastrocnemius and plantar muscles for 60 days beginning on day 7, at which time CMAP amplitudes were depressed to 18 and 4%, respectively, of values observed in the non-impaired contralateral muscles. A time-dependent recovery of CMAP amplitude was observed in both saline- and BoNT/A-injected mice, however, the values in the latter group were significantly greater at times ≥21 days following CCI. Moreover, in saline-injected mice, the ratio of the Hoffmann reflex (H-reflex) to the direct motor (M) response increased from day 14 to 28 following CCI, consistent with the development of hyperreflexia [126]. However, a single injection of BoNT/A in the plantar region on day 5 counteracted this abnormal condition.

Cobianchi et al. [32] were the first to report that BoNT/A can increase axonal regeneration after nerve crush. These authors also extended earlier finding by Marinelli et al. [120] that BoNT/A causes enhanced proliferation of the dedifferentiated repair-promoting phenotype of Schwann cells after nerve crush. This was indicated by findings that BoNT/A increased the expression of S100β and GFAP, both of which are upregulated in Schwann cells after nerve injury [70,72]. Proliferation of repair Schwann cells is a crucial event for successful nerve regeneration [70,72,127] and may be the primary mechanism for the regenerative potential of BoNT/A [32]. While not directly examined in this study, the authors suggested that BoNT/A-mediated inhibition of neurotransmitters such as glutamate, substance P, and calcitonin gene-related peptide (CGRP) [113,114,115] may also play a role in the regenerative actions of BoNT/A, possibly by limiting the inflammatory phase of the regeneration process.

### 3.2. BoNT/A Enhances Recovery from PNI in Rat Femoral Nerve Transection-Repair Model

A regenerative effect of BoNT/A was demonstrated in the rat femoral nerve axotomy model by Irintchev et al. [30]. Nerve injury was elicited by cutting the femoral nerve 7 mm above its saphenous/quadriceps bifurcation, and the cut end of the proximal stump was incubated for 30 min in a 10 µL solution containing 100 U/mL BoNT/A (incobotulinumtoxinA, Xeomin^®^). After toxin exposure, the nerve was rinsed with saline to remove excess BoNT/A, and the proximal and distal cut ends were coapted using epineural sutures. The actions of BoNT/A were assessed on functional recovery by monitoring alterations in the foot–base angle (FBA) and step length ratio (SLR) during walking trials on a narrow beam. Injury to the femoral nerve led to an increase in the FBA due to abnormal external rotation of the heel elicited by underlying dysfunction of the quadriceps muscle. Femoral nerve injury also caused an increase in the SLR (ratio of control/injured limbs), due to the compensatory shorter step lengths required for the injured limb to support the rat’s body weight [128]. A single 30-min exposure of the proximal nerve stump to BoNT/A prior to coaptation led to a marked acceleration in the FBA × SLR recovery index; complete recovery was observed at ~3.5 weeks after BoNT/A treatment, whereas the recovery index was abnormal even after 20 weeks in animals whose femoral nerves were treated with vehicle (saline plus 0.1% bovine serum albumin).

BoNT/A was also evaluated by Irintchev et al. [30] by examining the ability of the neurotoxin to antagonize axotomy-induced alterations in the density of excitatory and inhibitory perisomatic synaptic terminals on spinal motor neurons in the femoral motor nucleus. The authors examined the actions of BoNT/A on proliferation of microglia and macrophages using established markers. These consisted of vesicular GABA transporter (VGAT), vesicular glutamate transporter 1 & 2 (VGLUT1, VGLUT2), choline acetyl transferase (ChAT), and ionized calcium binding adaptor molecule 1 (Iba1). At 1 week after axotomy, BoNT/A increased the density of VGAT+ and ChAT+ terminals, decreased the density of VGLUT1+ but not VGLUT2+ terminals, and had no effect on the density of Iba1+ cells (presumed to be microglia). When examined at 2 months after nerve injury, the BoNT/A effects on VGAT+ and VGLUT1+ terminals were no longer observed but the preservation of ChAT+ terminals persisted. It was suggested that the early actions of BoNT/A may play a role in enhancing nerve regeneration by mitigating the increased excitability of the axotomized motor neurons. The sustained increase in cholinergic innervation, as indicated by the persistent effect of BoNT/A on ChAT+ terminals, was suggested to play a more direct role by increasing motor neuron output via activation of M2 muscarinic receptors to accelerate recovery of function. In neonatal spinal cord slices, M2 receptor activation has been shown to modulate membrane electrical properties such as lowering the rheobase, modifying the after hyperpolarization, and shortening the action potential duration [129].

### 3.3. Efficacy of Intramuscular BoNT/A Injected in the Contralateral Hindlimb

Lima et al. [130] demonstrated that BoNT/A can enhance nerve regeneration when injected in the gastrocnemius muscle contralateral to the one subjected to nerve transection. This study was performed in rats in which the right tibial nerve was transected at the midpoint between its origin at the level of the sciatic nerve trifurcation and its destination in the gastrocnemius muscle. The nerve was immediately repaired by end-to-end coaptation and endoneurial suturing to allow for reinnervation. Twenty µL of BoNT/A complex (abobotulinumtoxinA, Dysport^®^) containing 16 U/kg was injected into the left (contralateral) gastrocnemius muscle on the day of injury. In contrast to the studies reviewed thus far, this dose of BoNT/A produced muscle paralysis that lasted for 8 weeks. Improvements in nerve regeneration were assessed from measurements of axonal densities and CMAP characteristics at 12 weeks after BoNT/A treatment. Functional recovery was evaluated by examining walking track performance at the end of 12 weeks.

Walking track analysis was quantified by the tibial function index (TFI) in which 0 represents normal function and −100 denotes complete dysfunction. In rats subjected to nerve transection without repair, the TFI ranged from −57 to −68 and showed no improvement with time over the 12-week duration of the study. Rats in which the tibial nerve was repaired immediately after transection also exhibited poor TFI values (range −39 to −42), although the TFI values were slightly improved compared to rats whose tibial nerve was transected without repair. In contrast, rats treated as above but injected with BoNT/A in the contralateral gastrocnemius muscle had TFI values in the normal range on the surgically repaired side. Consistent with improvement in TFI, the amplitude and latency of CMAPs in this group of rats were also found to be in the normal range. In rats undergoing nerve transection and repair in the absence of BoNT/A, CMAP amplitudes were only 57% of control at 12 weeks in spite of having surgical repair performed immediately after transection.

Axonal density, measured 3 mm distal to the neurorrhaphy site, was found to be significantly higher in the axotomized-repaired tibial nerves than in control (sham operated) nerves and was not further increased by injection of BoNT/A in the contralateral gastrocnemius muscle. The authors concluded that the greater functional recovery in rats with contralateral BoNT/A injection does not reflect an increase in the total number of nerve fibers regenerated, but instead, may indicate a higher accuracy of reinnervation in the BoNT/A-treated group. The mechanism responsible for the improved outcome in the BoNT/A-injected rats was suggested to be a compensatory remodeling of the motor cortex in response to the transient BoNT/A-mediated paralysis of the contralateral limb, a mechanism similar to that proposed for improvement in facial symmetry in humans.

Although the injury model of Lima et al. [130] was similar to that of Irintchev et al. [30] (axonal transection followed by neurorrhaphy), the experimental approach of the former study differed from that of the latter in the following manner: (1) exposure to BoNT/A was by intramuscular injection, rather than by bathing of the proximal nerve stump; (2) the toxin was delivered to the gastrocnemius muscle of the contralateral hind limb, rather than to the injured limb and (3) the dose of BoNT/A was sufficient to cause transient paralysis of the hindlimb musculature, rather than being sub-paralytic. The use of BoNT/A to paralyze a normal limb in order to elicit improvement in an impaired limb is a form of constraint-induced movement therapy (CIMP). CIMT is considered to be an effective rehabilitation strategy for treatment of hemiparesis following stroke, cerebral palsy, traumatic brain injury, and multiple sclerosis [131]. Potential advantages of using BoNT/A to elicit constraint is that it does not require voluntary compliance and it removes the need for oversight by the therapist.

The regenerative actions of BoNT/A are summarized in Table 1. BoNT/A was able to improve the outcome of injuries to peripheral nerves following CCI, crush and nerve transection followed by repair. Improvements were assessed by both functional outcomes such as walking track performance, as well as by alterations in axonal densities, biomarkers, and electrophysiological parameters. It should be noted that in most cases only a single dose and route were used, which differed among the studies. Although the results are encouraging, the lack of a standard approach makes it somewhat difficult to gauge the true potential of BoNT/A for accelerating recovery from PNI and for increasing the accuracy of reinnervation.

### 3.4. BoNT/B Improves Neuropathic Pain but Does Not Accelerate Functional Recovery

Since BoNT/B is the only other approved pharmaceutical botulinum neurotoxin in the U.S., and a small percentage of patients may become resistant to BoNT/A treatment, Finocchiaro et al. [132] examined the effects of BoNT/B on nerve regeneration rates. In common with BoNT/A, BoNT/B also inhibits neurotransmitter release by interfering with the formation of the SNARE complex; in this case by cleaving vesicle associated membrane protein 1/2 (synaptobrevin 1/2, VAMP 1/2) [92].

BoNT/B (5 or 7.5 pg/mouse of 150 kDa neurotoxin) was administered to mice by intraplantar injection, and its ability to reduce mechanical allodynia and accelerate functional recovery from CCI was assessed 3 days after injury [132]. Mechanical allodynia was determined by measuring the withdrawal threshold of the injured vs. contralateral hind- paws to punctate mechanical stimuli using an automated von Frey apparatus. BoNT/A was included in some of the studies as a positive control.

CCI increased the sensitivity of the ipsilateral (injured) paw to mechanical stimuli as indicated by reduction of the mechanical nociceptive threshold to approximately 50% of the level of the contralateral paw. Recovery was slow, and residual hypersensitivity continued to be observed even at the last time point examined (101 days after CCI). A single intraplantar injection of BoNT/B (5 or 7.5 pg/paw) was able to counteract CCI-induced allodynia, and withdrawal thresholds were restored to nearly 80% of the contralateral, uninjured paw after one day with either dose of BoNT/B. This was similar to results obtained with BoNT/A (15 pg/paw) in the study of Marinelli et al. [120] described earlier.

However, unlike BoNT/A, intraplantar injection of BoNT/B at either 5 or 7.5 pg/paw had no effect on recovery of function as determined by the inability of BoNT/B to correct the asymmetric body weight distribution favoring the non-injured hindlimb and abnormal walking pattern caused by CCI. In fact, walking track performance was actually degraded by BoNT/B. To shed light on the absence of a pro-regenerative effect of BoNT/B, the sciatic nerve proximal to the CCI was stained for neurofilaments using NF200 polyclonal antibody 7 days after CCI and examined by confocal microscopy. Consistent with the absence of functional recovery, neurofilaments in BoNT/B-treated mice were found to be highly disorganized, resembling the cytoskeleton of mice undergoing CCI and treated only with saline. In contrast, neurofilaments of BoNT/A-treated mice were well organized at day 7, resembling the cytoskeletal organization of control mice. The lack of improvement in cytoskeletal structure was suggested to contribute to the absence of functional recovery with BoNT/B treatment.

Additional immunostaining was performed to shed light on the differential actions of BoNT/A and BoNT/B following CCI. BoNT/A produced a marked elevation in the expression of markers for proliferation of mast cells (chymase 1), peripheral macrophages (cluster of differentiation 11b; CD11b), and repair Schwann cells (GFAP). These cells work in a coordinated fashion to remove myelin fragments and axonal debris to facilitate nerve regeneration following peripheral nerve injury [34,133,134]. BoNT/B, on the other hand, reduced the proliferation of repair Schwann cells, had no effect on proliferation of macrophages and produced a less robust increase in mast cells (70% vs. 340%). The modest increase in mast cells, in the absence of an enhancement of macrophages and repair Schwann cells, is also likely to contribute to the lack of a pro-regenerative effect of BoNT/B.

The antiallodynic effect of BoNT/B was suggested to involve retrograde transport of the toxin to the spinal cord and subsequent transcytosis to astrocytes. Upon internalization into astrocytes, BoNT/B would be able to inhibit the release of glutamate, one of the principal neurotransmitters associated with pain [113]. Although direct evidence for this mechanism was not provided, it is noteworthy that cultured astrocytes have the ability to release glutamate in a calcium-dependent fashion, which can be inhibited by clostridial neurotoxins, albeit at high concentrations [135].

### 3.5. Effect of BoNT/A Preconditioning on Reinnervation

It has long been known that regeneration of an injured peripheral nerve can be accelerated if the same nerve has been subjected to an earlier injury within a narrow time interval (generally 1–2 weeks) before the second injury [136]. The enhancement is thought to occur because the initial injury (conditioning lesion) creates a pro-regenerative state by increasing protein synthesis and initiating other early neuronal changes in the soma. It was suggested that if a second injury occurs during the time that the regeneration program has already been activated, axonal regeneration and functional recovery should be accelerated. McQuarrie and Grafstein demonstrated this phenomenon on the mouse sciatic nerve nearly 50 years ago [137]. The authors produced the initial injury by crushing the sciatic nerve near the popliteal fossa. After 2 weeks, a second lesion was produced in which a 1 cm section of nerve above the crush site was removed, and new axonal outgrowth from the proximal stump was measured for 16 days for evidence of accelerated axonal growth. Removal of the 1 cm nerve segment ensured that the changes observed were not caused by alterations in support cells, but instead, reflected alterations in the neurons proximal to the conditioning lesion. The authors found that the rate of axonal growth was increased by nearly 30% in mice subjected to a conditioning injury relative to growth rates in mice undergoing nerve excision only.

In a subsequent study, McQuarrie et al. [138] examined the effect of conditioning lesions under somewhat different experimental conditions from their earlier study: the conditioning lesion was elicited by transection rather than crush, the site of the conditioning injury was more distal (near the ankle), and the test injury was elicited by crushing rather than sectioning the sciatic nerve, thus allowing the emerging axons to grow into the distal nerve stump. As before, the conditioning and test lesions were performed 2 weeks apart. Functional nerve outgrowth was determined by lightly pinching the nerve with fine forceps and proceeding proximally in 2 mm increments from the most distant outgrowth until a withdrawal reflex was elicited. Using the above paradigm, the conditioning injury elicited a significant increase in the regeneration rate (23%) relative to rates observed in animals receiving only the test injury. These were considered to be more realistic conditions, since in the earlier study [137] the regenerated nerves grew into connective tissue rather than into the distal nerve stump.

Although the principle of enhanced regeneration after a conditioning lesion is of considerable interest, it is not readily translatable to the clinical setting due the invasive nature of the procedure and the requirement for the lesion to occur prior to nerve injury. However, if chemodenervation by BoNT/A is able to serve as a nonsurgical alternative for the conditioning lesion, it may be possible to exploit this phenomenon for achieving accelerated nerve growth and presumably a more satisfactory outcome after nerve injury and repair. Data consistent with this expectation were recently provided by Franz et al. [139]. The authors injected 0.25 units of BoNT/A (onabotulinumtoxinA, Botox^®^) unilaterally into the triceps surae muscle group in mice. BoNT/A produced a marked paralysis of the injected hindlimb as revealed by impairment of the toe spread reflex and prominent ventroflexion of the toes. Paralysis lasted for 3 weeks, with peak effects occurring 3 days after the BoNT/A injection. At day 7, when the BoNT/A-mediated paralysis was still close to maximum, the tibial nerve on the injected side was crushed using jeweler’s forceps with the aim of severing all axons. One week after nerve crush, a segment of tibial nerve 10–12 mm distal to the crush site was examined for the presence of myelinated axons. BoNT/A conditioning enhanced the number of myelinated axons almost 2-fold relative to vehicle-injected controls undergoing crush.

The authors also investigated the effect of BoNT/A followed by crush on the number of retrograde labeled neurons 1 week and 4 weeks after injury. Tibial nerves were transected 10 mm distal to the crush site, and Fluoro-Ruby dye was applied to the nerve with gel foam. In each case, motor neurons were observed 1 week after tracer application. For mice in the 1-week crush group, BoNT/A conditioning produced a significant increase (39%) in the number of motor neurons visualized by Fluoro-Ruby. No difference was observed in the 4-week group, since motor neuron staining in both the BoNT/A- and vehicle-injected mice had returned to pre-lesion values. Although the benefit of BoNT/A was transient, it would still be of value to increase regenerating rates, especially when injuries are more serious or involve longer reinnervation paths [4,23].

In contrast to the study of Franz et al. [139], several authors have not been able to demonstrate a beneficial effect of BoNT/A conditioning on a subsequent test injury as exemplified by the study of Brown and Hopkins [140]. These authors examined the effect of a crude preparation of BoNT/A (2 µL of a 10 µg/mL solution) injected in the soleus muscle of mice 12 days prior to crushing of the soleus nerve; the test lesion was made 1 mm from entry of the soleus nerve into the muscle. This dose of BoNT/A corresponds to 10 times the mouse LD50 dose if injected intraperitoneally. Brown and Hopkins [140] reported that BoNT/A was unable to accelerate recovery of neurally elicited muscle tension. However, as noted by Franz et al. [139], crushing the nerve so close to the muscle may not have provided a sufficiently long nerve segment to detect a BoNT/A-mediated effect. In addition, reinnervation was assessed by restoration of muscle tension and motor unit numbers, both of which are influenced by the time required for the occurrence of synaptogenesis in addition to regeneration rates. A more direct measure of nerve regeneration would have been preferable, since it is not clear which process is rate limiting under the experimental conditions used by Brown and Hopkins [140]. Based on the foregoing, further research on the use of BoNT to elicit conditioning injury such as optimization of dose, serotype/subtype, interval between toxin injection, and injury will be needed to evaluate the potential benefits of BoNT conditioning for accelerating nerve regeneration.

Although a number of candidate therapeutic agents, exclusive of BoNT/A, have been found to improve peripheral nerve regeneration, few studies have investigated the potential synergy of drug combinations. A small pilot study in mice was recently published by Odorico et al. [141], in which the authors examined the combined actions of nimodipine and BoNT/A. Nimodipine is a Ca^2+^ channel blocker used in adults to reduce cerebral vasospasm following subarachnoid hemorrhage. Nimodipine has also been reported to improve the outcome of PNI in rats and mice. The authors evaluated the drugs individually and in combination for their ability to enhance the outcome of PNI in rats subjected to tibial nerve transection, followed immediately by neurorrhaphy. Recovery from the PNI was assessed using functional, electrophysiological, and histologic criteria. Surprisingly, the data indicated that the combination of nimodipine and BoNT/A did not lead to improved regeneration, and in some assays actually led to poorer results than those observed with single drug therapy. This finding suggests that a better understanding is needed of how these drugs affect nerve regeneration in order to obtain synergy with drug combinations. In the absence of such information, it is difficult to optimize dose, interval, and sequence of administration for achieving a favorable outcome.

### 3.6. Potential Mechanisms of BoNT/A-Induced Acceleration of Nerve Regeneration

Various mechanisms have been proposed for the regenerative action of BoNT/A following injury to peripheral nerves, for example, inhibition by BoNT/A of proinflammatory neurotransmitters, which may limit the inflammatory phase of the regeneration process [32]. Two proposed mechanisms have received reasonable experimental support and will be considered here: enhancement in the normal response of Schwann cells to injury [32,49,120] and increase in blood flow and angiogenesis to support the regenerating nerve [1,34].

#### 3.6.1. BoNT/A Action Mediated via Interaction with Schwann Cells

As mentioned earlier (Section 3.1), Schwann cells play a critical role in peripheral nerve regeneration [82,89,90,91,127]. In response to nerve injury, Schwann cells undergo marked changes in gene expression, leading to dedifferentiation, proliferation, and conversion to the repair phenotype. Repair Schwann cells produce neurotrophic factors to provide a supportive environment for axon regeneration and form guidance tracks (bands of Büngner) for directing the regenerating axons to their appropriate targets [72]. Both Marinelli et al. [120] and Cobianchi et al. [32] suggested that the regenerative effect of BoNT/A after neve crush or CCI was primarily due to the ability of the neurotoxin to augment the normal response of Schwann cells to injury. Consistent with this proposal, the authors found that exposure to BoNT/A led to a marked increase in Schwann cell proliferation as evidenced by increases in the Schwann cell markers S100β and GFAP. In addition, using Schwann cells cultured from rat sciatic nerve, Marinelli et al. [142] found that a low concentration of BoNT/A (10 pM) inhibited ACh release from Schwann cells and increased their rate of proliferation. They suggested that inhibition of ACh release by BoNT/A may be a key trigger for enhancing the transition of Schwann cells to the repair phenotype after injury, based on earlier data that activation of M2 muscarinic receptors by ACh represses Schwann cell proliferation and contributes to their transition to the differentiated myelinating phenotype [143,144].

While this mechanism is plausible, it has not yet been demonstrated in vivo that release of ACh from Schwann cells governs their transition from repair to myelinating phenotype, and further, that BoNT/A at the doses used was able to inhibit Schwann cell ACh release sufficiently to elicit their dedifferentiation to the repair phenotype.

#### 3.6.2. BoNT/A Action Mediated via Improved Blood Flow and Increased Angiogenesis

In addition to its action on Schwann cells, BoNT/A may also accelerate nerve regeneration by improving blood flow and increasing angiogenesis. Nerve injury not only damages axons, myelin, and connective tissue but also destroys the integrity of the local vasculature. Early in the recovery phase, there is a significant increase in the mean radius of endoneurial blood vessels, most prominently at the site of injury. The increase in radius reaches its maxima at 1 week after nerve crush, during which time the number of blood vessels remains unchanged [145]. This early phase coincides with recruitment of macrophages and removal of axonal and myelin debris resulting from Wallerian degeneration [1]. The increase in blood flow promotes healing by bringing more nutrients, oxygen, and growth factors to the injured nerve [146].

Accordingly, we propose that one of the pro-regenerative actions of BoNT/A may be to further increase blood flow to the site of injury. Support for this mechanism comes from finding that BoNT/A can increase blood flow in frostbite [38], vasospastic disorders such as Raynaud’s phenomenon [39], and ischemic hand trauma [40]. The mechanism of BoNT/A mediated dilation of blood vessels remains to be determined, especially in PNI. However, it has been proposed that BoNT/A causes vasodilation by reducing arterial smooth muscle calcium sensitization and by increasing the activity of nitric oxide synthase (NOS), leading to increased production of the vasodilator, nitric oxide [147]. Inhibition of norepinephrine release from sympathetic vasomotor neurons may also contribute to the vasodilatory action of BoNT/A. However, its importance to this process is uncertain due to the high concentrations of BoNT/A needed to inhibit norepinephrine release [68]. In addition, adrenergic innervation in mammalian peripheral nerves is confined to blood vessels in the epineurium and perineurium but is absent from endoneurial blood vessels [148].

An increase in angiogenesis is responsible for the delayed increase in blood flow after injury [1]. This phase develops more slowly, with the peak response in rat sciatic nerve occurring 6 weeks after nerve crush [145]. Newly formed blood vessels not only provide increased blood flow to the regenerating nerve, but also serve to guide the migration of Schwann cells. Cattin et al. [34] studied angiogenesis in transected rat sciatic nerve. Transection caused retraction of the nerve stumps proximal and distal to the cut, leading to a gap of several millimeters. If the gap is sufficiently narrow, the stumps can be rejoined by spontaneous formation of a nerve bridge, thus providing a pathway for regeneration. Cattin et al. [34] demonstrated that resident macrophages secrete VEGF-A in response to hypoxic conditions within the nerve bridge, which leads to recruitment of endothelial cells and vascularization of the bridge. The newly formed blood vessels in the bridge orient in the direction of the regeneration path and serve to guide Schwann cells and regerating axons across the bridge to reach the distal stump and reinnervate target tissues.

More recently Fornasari et al. [149] investigated whether a similar mechnism also applies for regeneration across nerve conduits used to repair large nerve gaps in rat median nerves transected and repaired with 10 mm chitosan conduits. In spite of the much larger gap, the authors found that that angiogenesis played a similar role in nerve conduits as it did in nerve bridges by providing a vascularized pathway for the migration of newly formed Schwann cells to enable nerve regeneration.

Although evidence that angiogenesis contributes to the regenerative action of BoNT/A after PNI is indirect, there are a number of studies that implicate BoNT/A-mediated angiogenesis in improving the outcome of wound healing that involves grafts, flaps or tissue expansion [35,36,37]. It is therefore reasonable to assume that enhanced angiogenesis by BoNT/A may also contribute to its regenerative actions in recovery from PNI. Thus, Kim et al. [35] investigated the actions of BoNT/A on random cutaneus flaps in a rat model. Injection of BoNT/A (1.5 U) into the dermal layer of the flap led to improved tissue survival along with increases in the number and diameter of blood vessels in the flaps. Using RT-PCR, the authors also found that BoNT/A enhanced the expression of cluster of differentiation 31 (CD31), VEGF and NOS mRNA, consistent with the BoNT/A-mediated angiogenesis and vasodilation.

Similar results were reported by Park et al. [36] on flaps pretreated with BoNT/A 5 days prior to surgery. BoNT/A led to increases in tissue survival, angiogenesis and blood vessel diameter. RT-PCR and Western blotting revealed increased expression of the angiogenic cytokines cluster of differentiation 34 (CD34), VEGF and hypoxia inducible factor 1α (HIF-1α). Consistent with the above studies, Liu et al. [37] found that BoNT/A (1 U per injection site) improved the success of expanded skin surgery which was attributed to increased angiogenesis and vasodilation leading to improved blood flow.

## 4. Conclusions

PNI has a high rate of incidence and can result in long-term sensory, motor, and autonomic dysfunction. Although peripheral nerves have a high intrinsic rate of reinnervation, incomplete or unsatisfactory outcomes are common following injuries that are delayed, severe, occur close to the soma or compromise the endoneurial tubes that are necessary for the reinnervation process. A considerable number of candidate therapeutic agents have been examined to accelerate the rate of reinnervation such as corticosteroids, carnitine, FK-506, nerve growth factor, IGF-1, VEGF, TGF-β1, BDNF, and 4-AP. Non-pharmacological methods such as electrical stimulation [150] and extracorporeal shock wave therapy have also been investigated, the latter providing benefits similar to that of BoNT/A [151]. In spite of the large number of candidates tested, there are no approved pharmacological treatments in clinical use for PNI capable of accelerating the rate and accuracy of nerve regeneration. Problems include poor stability, short duration of action, and numerous off-target effects.

BoNT/A, in spite of being a highly potent paralytic neurotoxin, has exquisite selectivity, remarkable stability, a relatively rapid onset and a long duration of action. In addition, BoNT/A is able to gain access to the cell body of peripheral neurons by retrograde transport and mitigate neuropathic pain. Recent studies reviewed in this manuscript have revealed some less well-known actions of BoNT/A including an ability to accelerate nerve regeneration following CCI, nerve crush and axotomy. These actions have been attributed primarily to increased proliferation of Schwann cells and conversion to the repair phenotype as well as activation of mast cells and macrophages. Thus far, increases in reinnervation rates and of functional recovery have only been demonstrated for BoNT/A; BoNT/B was found to have similar antinociceptive actions but to lack the regenerative properties of BoNT/A. Based on the promising work in mice and rats, it would be of considerable interest to extend these studies to larger animals where the magnitude of change seen in rodents may have more profound consequences due to their longer peripheral nerves and slower rate of spontaneous regeneration. In addition, new preclinical studies should include dose-response studies and long-term evaluation of functional recovery; these were generally not pursued in these proof-of-concept studies. Further, all the studies reported herein were performed using BoNT/A1 and BoNT/B1, which are also the subtypes in clinical use. Comparatively little is known of the clinical potential of other subtypes of BoNT/A and BoNT/B [44,119]. Differences among the subtypes have been observed in rates of onset, potency, persistence, diffusion from injection site, and retrograde transport [95,97,99]. A better understanding of these subtypes may reveal additional candidates for improving the outcome of injuries to peripheral nerves.

## Figures and Tables

**Table 1:** Regenerative Actions of BoNT/A in Rodent Models.

Ref.	Nerve Injury	Species	Formulation & Dose	Route	Major Assessments	Primary Finding	Proposed Mechanism
[120]	CCI ^1^	Mouse	150 kDa BoNT/A; 15 pg/paw	Intraplantar injection	Weight bearing; walking track analysis	Normalization of weight bearing; accelerated recovery of SSI ^2^	Proliferation of repair Schwann cells
[32]	Nerve crush	Mouse	150 kDa BoNT/A; 15 pg in 2 µL saline	Intraneural injection at crush site	Pinch test; CMAP ^3^; CNAP ^4^; nerve fiber number and density	Increased rate of regeneration of myelinated nerves	Proliferation of repair Schwann cells; reduced inflammation
	CCI ^1^	Mouse	15 pg in 20 µL saline	Intraplantar injection			
[30]	Transection/repair of femoral nerve	Rat	IncobotulinumtoxinA (Xeomin^®^); 100 U/mL ^5^ for 30 min	Incubation of proximal nerve stump	Walking track analysis; histochemical staining	Marked acceleration in recovery of ^6^ FBA × SLR index	Preservation of cholinergic input to femoral motor neurons
[130]	Transection of tibial nerve with and without repair	Rat	AbobotulinumtoxinA (Dysport^®^); 16 U/kg	Intramuscular injection in contralateral gastrocnemius muscle	Walking track analysis; EMG; axonal density	Accelerated recovery of walking track performance in rats undergoing nerve transection with immediate repair	Adaptation by CNS in response to muscle weakness in contralateral limb

^1^ CCI = chronic constriction injury; ^2^ SSI = sciatic static index; ^3^ CMAP = compound muscle action potential; ^4^ CNAP = compound nerve action potential; ^5^ U = mouse intraperitoneal lethal dose50 (LD50) units; ^6^ FBA × SLR = foot-base angle × step length ratio.
